# Macrothrombocytopenia with leukocyte inclusions in a patient with Wilson disease: a case report and literature review

**DOI:** 10.1186/s12920-024-01960-1

**Published:** 2024-07-17

**Authors:** Shaoze Lin, Jianling Cai, Yuxuan Huang, Hongxing Chen, Meidie Yu, Dongqing Zhang, Zhanqin Huang

**Affiliations:** 1 Department of Hematology, the First Affiliated Hospital of Shantou University Medical College, Shantou, 515041 Guangdong P.R. China; 2 Department of Pediatrics, the First Affiliated Hospital of Shantou University Medical College, Shantou, 515041 Guangdong P.R. China; 3 Shantou University Medical College, Shantou, 515041 Guangdong P.R. China; 4 Department of Laboratory Medicine, the First Affiliated Hospital of Shantou University Medical College, 57 Changping Road, Shantou, 515041 Guangdong P.R. China; 5 Department of Pharmacology, Shantou University Medical College, Shantou, 515041 Guangdong P.R. China

**Keywords:** Wilson disease, Thrombocytopenia, Leukocyte inclusions, Giant platelets, Heterozygote

## Abstract

**Background:**

Wilson disease (WD) is an autosomal recessive disorder caused by homozygous or compound heterozygous mutations in *ATP7B*. Clinical manifestations primarily involve liver and nervous system lesions, with rarely observed hematologic manifestations.

**Case presentation:**

In the present case, a patient with WD presented with thrombocytopenia, giant platelets, and Döhle-like cytoplasmic inclusions in the leukocytes. Initially, the May–Hegglin anomaly was considered; however, whole-exome sequencing did not reveal any mutation in the *MYH9* gene but a heterozygous mutation was found in (C.2804 C > T, p.T935M) in the *ATP7B* gene. After two years, the patient developed tremors in his hands, lower limb stiffness, and foreign body sensation in the eyes. Additionally, Kayser–Fleischer rings in the corneal limbus were detected by slit-lamp examination. Copper metabolism test indicated a slight decrease in serum ceruloplasmin. Transmission electron microscopy revealed that the inclusion bodies of leukocytes were swollen mitochondria. Mass spectrometry analysis showed that the copper levels were almost 20-fold higher in the leukocytes of the patient than in those of the control group. Based on the Leipzig scoring system, a diagnosis of WD was confirmed. Zinc sulfate treatment ameliorated the patient’s symptoms and enhanced platelet, serum ceruloplasmin, and albumin levels.

**Conclusions:**

In conclusion, this case represents the first documented instance of WD presenting as thrombocytopenia, giant platelets, and Döhle-like cytoplasmic inclusions in the leukocytes. Excessive cellular copper accumulation likely underlies these findings; however, understanding precise mechanisms warrants further investigation.

## Background

Wilson disease (WD) is an autosomal recessive disorder resulting from *ATP7B* gene dysfunction due to mutation, leading to copper accumulation in various tissues, primarily in the liver and brain [1]. The spectrum of manifestations varies, encompassing liver cirrhosis, brain neuronal degeneration, Kayser–Fleischer (K-F) rings in the corneal limbus, kidney damage, and hemolytic anemia [2–4]. WD is often misdiagnosed because of the diverse clinical presentations.

In this study, we present a case of a middle-aged woman exhibiting thrombocytopenia, giant platelets, and Döhle-like cytoplasmic inclusions in leukocytes. Whole-exome sequencing (WES) revealed a heterozygous *ATP7B* gene mutation. Two years after this finding, the patient developed hand tremors, lower limb stiffness, and foreign body sensation in the eyes. We questioned whether these symptoms were associated with WD. To explore this possibility further, we performed copper metabolism analysis, slit-lamp examination, magnetic resonance imaging (MRI), transmission electron microscopy (TEM) observation, and leukocyte copper content detection.

## Case presentation

**Fig. 1:**
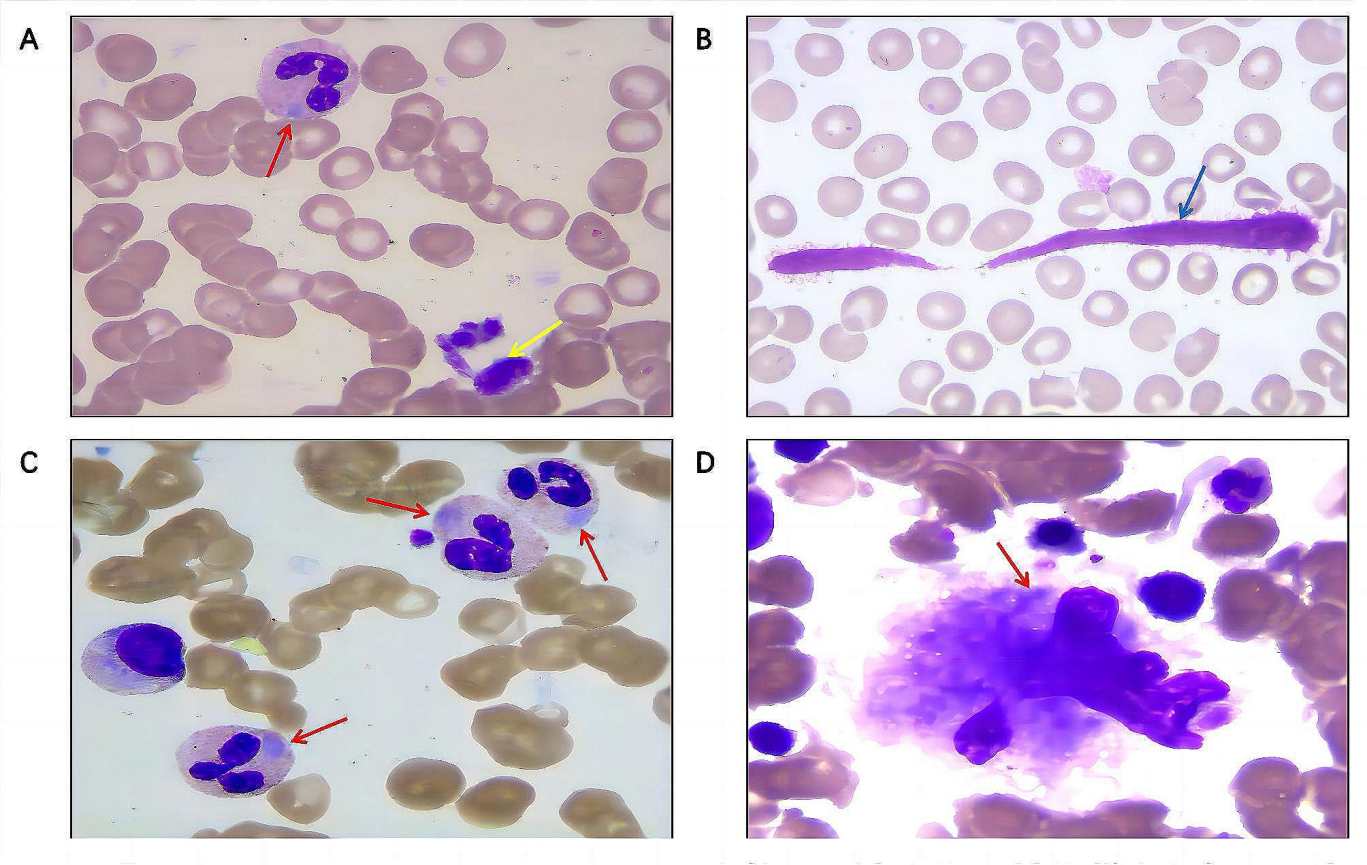
Peripheral blood and bone marrow smears of the patient. (**A**) Larger platelet (yellow arrow) and Döhle-like cytoplasmic inclusion (red arrow) in neutrophils on the peripheral blood smear. (**B**) Giant platelet (blue arrow) on the peripheral blood smear. (**C**) Döhle-like cytoplasmic inclusions in neutrophils (red arrow) on the bone marrow smear. (**D**) Döhle-like cytoplasmic inclusions in a megakaryocyte (red arrow) on the bone marrow smear (Wright’s stain, ×400, all)

**Fig. 2:**
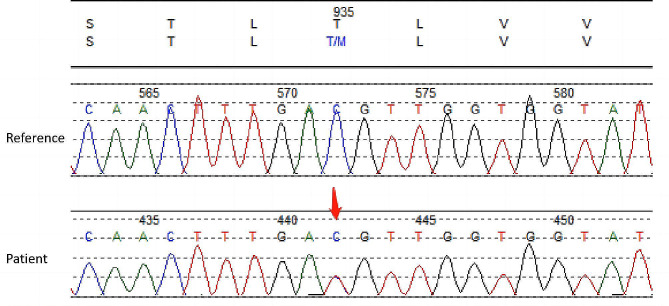
Sequence chromatogram showing the heterozygous nucleotide sequence of c.2840 C > T. Red arrow indicates the mutation site

In June 2021, a 48-year-old woman presented at our hospital with a 2-year history of easy bruising. Complete blood counts were normal; however, thrombocytopenia was observed (white blood cell count: 3.88 × 10^9^/L; red blood cell count: 3.80 × 10^12^/L; hemoglobin: 115 g/L; platelet count: 65 × 10^9^/L). Other laboratory parameters, including antiplatelet and antinuclear antibodies, thromboaggregation function, von Willebrand factor antigens, prothrombin time, and activated partial thromboplastin time, were within normal limits. Liver function test results were mainly normal, except for reduced albumin levels (37.84 g/L; normal range: 40.0–55.0 g/L). Peripheral blood smear revealed thrombocytopenia, with large and giant platelets and Döhle-like cytoplasmic inclusions in the leukocytes. Bone marrow smears showed easily observable Döhle-like cytoplasmic inclusions in neutrophils and megakaryocytes, with degenerative cells being common (Fig. 1). Initially, the May–Hegglin anomaly (MHA) was provisionally diagnosed. However, WES showed no mutation in the *MYH9* gene but indicated a heterozygous mutation in the twelfth exon of the *ATP7B* gene (C.2804 C > T, p.T935M), altering threonine at codon 935 to methionine. The vatiant was verified by Sanger sequencing (Fig. 2). This variant is a hotspot mutation in *ATP7B*. However, the patient lacked typical WD symptoms, so a diagnosis of WD was not made at that time. Dexamethasone therapy (10 mg/dose, tid, p.o) was initiated but showed a poor response, with platelet counts fluctuating within the range 62–68 × 10^9^/L. No further treatment was administered.

In March 2023, the patient returned to our hospital with extremity ecchymosis, hand tremors, lower limb stiffness, and foreign body sensation in the eyes. Automatic blood cell analysis revealed a platelet count of 46 × 10^9^/L. Peripheral blood and bone marrow smears were similar to those from two years prior. Copper metabolism assessment showed serum ceruloplasmin, serum copper, and 24-hour urine copper levels of 23 (normal range: 25–45) mg/dL, 1161.4 (normal range: 800.0–1550.0) µg/L, and 21.1 (normal range: 15.0–60.0) µg/24 h, respectively. Liver function tests indicated decreased albumin levels (35.97 g/L; normal range: 40.00–55.00 g/L).

**Fig. 3:**
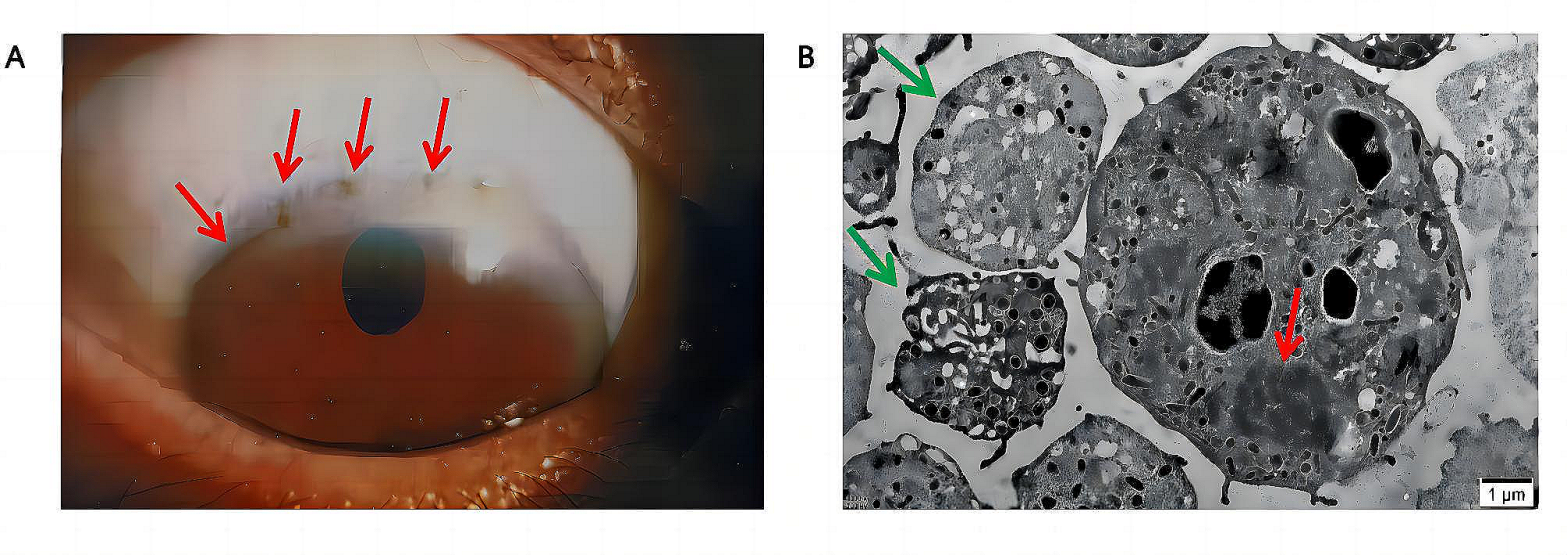
(**A**) Brown-spotted, arc-shaped copper deposits observed in the corneal limbus (red arrow). (**B**) Large platelets with adequate α granules and dense particles observed via Transmission electron microscopy (TEM) (green arrow). Swollen mitochondria in leukocytes observed by TEM (red arrow)

Brain MRI displayed minimal T1W1 low-intensity signal and T2W1 high-intensity signal in both ventricular anterior corners. Abdominal MRI showed multiple liver nodules with prolonged T1 and T2 signals. Slit-lamp examination revealed K-F rings in the corneal limbus (Fig. 3A). TEM showed abundant α particles and dense particles in the giant platelets, along with irregularly expanded and fuzzy inclusion bodies (mitochondria) with broken cristae in leukocytes (Fig. 3B).

Leukocyte copper content, measured using inductively coupled plasma mass spectrometry, was 1.41 fg/cell for the patient and 0.07 ± 0.04 fg/cell for the control group (*n* = 10), representing nearly a 20-fold increase in the patient.

Based on these findings, the patient was diagnosed with WD according to the Leipzig scoring system proposed by an international group for the study of WD [5]. The patient’s Leipzig score was 4 points (K-F rings present, score 2; neurological symptoms, score 1; and only one chromosome detected, score 1). Therefore, the patient was prescribed the full dose of zinc sulfate (60 mg/dose, tid, p.o.) to treat WD. After 6 months of follow-up, they showed substantial improvement in their hand tremors, lower limb stiffness, ocular discomfort, and limb ecchymosis symptoms. Laboratory tests revealed a platelet count of 78 × 10^9^/L, serum ceruloplasmin concentration increase from 23 mg/dL to 25 mg/dL, and serum albumin level rise from 35.97 g/L to 42.3 g/L.

## Discussion and conclusions

WD arises from homozygous or compound heterozygous mutations in *ATP7B*, a gene encoding a copper-transporting P-type ATPase [6, 7]. Dysfunction in *ATP7B* leads to hepatic copper accumulation and excessive nonceruloplasmin-bound copper in the circulation, resulting in copper uptake and subsequent injury to the brain and kidneys, among other organs [8, 9]. Hepatic manifestations of WD range from asymptomatic transaminase elevation to fibrosis, cirrhosis, and acute liver failure. Heterozygotic carriers of faulty *ATP7B* may remain asymptomatic; however, 20% of cases exhibit copper metabolism abnormalities [10]. Notably, some heterozygotic mutation carriers show limb tremors or liver symptoms [11–13]. A single T935M heterozygotic mutation in the *ATP7B* gene of patients with WD has not been previously reported. In this case, a single T935M heterozygotic mutation in *ATP7B* was detected using WES and validated using Sanger sequencing. Furthermore, the patient gradually developed neurological symptoms and ocular discomfort, with K-F rings in the corneal limbus evident upon slit-lamp examination. Collectively, these findings confirmed the WD diagnosis. Moreover, effective zinc sulfate treatment corroborated the occurrence of excessive copper accumulation in the patient.

Hemolysis is an important hematologic manifestation of Wilson’s disease. An analysis of 321 case notes of patients with Wilson’s disease seen between 1955 and 2000 and one case seen in 1949 has revealed that 22 patients presented with a haemolytic crisis. [14]. Pop et al. reported that 8 children had Coombs-negative hemolytic anemia in the total analyzed cases with 51 WD patients (15.67%) [15]. Despite large number of cases have been reported, however, the mechanism of hemolysis in WD remains unclear. In addition, other hematologic manifestations, including leukopenia and thrombocytopenia have been rarely reported [16, 17].

In the present case, the patient exhibited thrombocytopenia, giant platelets, and Döhle-like cytoplasmic inclusions in leukocytes, similar to MHA. Interestingly, no *MYH9* gene mutation was found. Therefore, we questioned whether these abnormal hematological findings were associated with WD. Mass spectrometry revealed that leukocyte copper levels were almost 20-fold higher in the patient than in the control group. Previous studies have shown that, compared with patients with homozygous mutations, carriers exhibit slower copper deposition due to a partial decline in *ATP7B* gene function [18, 19]. In tissues, the toxicity of excess copper is attributed to its redox activity, which induces oxidative stress and results in lipid, protein, DNA, and RNA molecule damage [20]. At the subcellular level, mitochondria are highly sensitive to copper-induced toxicity, with excess copper causing mitochondrial impairment as well as DNA damage through reactive oxygen species production [21]. In the present case, Döhle-like cytoplasmic inclusions were clearly observed in leukocytes, with TEM indicating that the inclusion bodies of leukocytes were swollen mitochondria. Therefore, we postulated that chronic copper accumulation could induce toxic changes in the mitochondria of these cells.

The mechanism underlying thrombocytopenia in patients with WD is not fully understood. Thrombocytopenia with negative antiplatelet antibodies has been reported in children, attributed to hypersplenism and/or D-penicillamine therapy’s side-effects [22]. A few studies have indicated idiopathic thrombocytopenia as an initial sign in patients with WD [23, 24]. Our patient presented with thrombocytopenia, macroplatelets, and Döhle-like cytoplasmic inclusions in the megakaryocytes. They initially showed unresponsiveness to hormonal therapy, and zinc sulfate administration after WD diagnosis resulted in a platelet count increase from 46 × 10^9^/L to 78 × 10^9^/L, suggesting WD-related thrombocytopenia. However, the contribution of excess copper accumulation to these phenomena warrants further investigation. Because the patient declined a bone marrow biopsy, we will investigate the effects of copper exposure on megakaryocytes in vitro.

In summary, this case is the first report that WD patient was presented by thrombocytopenia, giant platelets, Dohle-like cytoplasmic inclusions in the leukocytes. Clinically, if patients have the above manifestations, genetic analysis should be performed to distinguish them from MHA to avoid misdiagnosis. A heterozygous carrier with a defective *ATP7B* gene might also show the similar symptoms of WD. The carriers with clinical symptoms should be paid more attention, and the progress of patients’ conditions should be monitored. The early diagnosis and timely treatment of WD are quite critical. This study has stimulated our interests in further exploring the pathophysiological mechanism of abnormal hematological changes caused by copper accumulation, especially how excessive copper affects megakaryocytes to induce thrombocytopenia with abnormal morphology will be the focus of our further research.

## Data Availability

The datasets used and analyzed during the current study are available from the corresponding author on reasonable request.

## Abbreviations

WD: Wilson disease
K-F: Kayser–Fleischer
WES: Whole-exome sequencing
MRI: magnetic resonance imaging
TEM: transmission electron microscopy
MHA: May–Hegglin anomaly
